# Exploring inbreeding depression in Brazilian Angus cattle population using pedigree and genomic data

**DOI:** 10.3389/fgene.2025.1613820

**Published:** 2025-06-09

**Authors:** Henrique A. Mulim, Gabriel S. Campos, Fernando Flores Cardoso, Hinayah Rojas de Oliveira

**Affiliations:** ^1^ Department of Animal Science, Purdue University, West Lafayette, IN, United States; ^2^ Brazilian Agricultural Research Corporation, Embrapa South Livestock Center, Bage, Brazil

**Keywords:** genetic diversity, genomic inbreeding, livestock genetics, quantitative traits, genomic selection, genomic relationship matrix, animal breeding

## Abstract

**Introduction:**

Inbreeding depression refers to the decline in performance caused by increased levels of inbreeding, which results from mating individuals with common ancestors. This study aimed to estimate inbreeding coefficients based on both pedigree and genomic information using six different metrics and evaluate, the inbreeding depression on different traits officially evaluated by the Brazilian Angus Association.

**Material and Methods:**

A total of 13,777 genotyped animals, imputed to a density of 78,837 SNPs, and 530,327 animals in the pedigree file, extending up to 17 generations, were used in the analysis. The inbreeding metrics evaluated included: pedigree-based inbreeding (F_PED_), genomic relationship matrix-based inbreeding (F_GRM_), observed vs. expected homozygosity (F_HOM1_), genotyped homozygosity (F_HOM2_), correlation between uniting gametes (F_UNI_), and runs of homozygosity (F_ROH_). Traits related to growth, conformation, meat quality, reproduction, resistance to ectoparasites, and heat stress were analyzed.

**Results:**

The results revealed a range of inbreeding coefficients, with inbreeding estimated using ROHs showing the highest values (0.13). The impact of inbreeding on various traits was predominantly negative, with significant inbreeding depression observed for traits such as hair coat. Some traits, such as intramuscular fat and birth weight, had positive associations with inbreeding, indicating a complex trait-specific relationship. Shorter ROH segments (<2 Mb) generally had smaller or beneficial effects compared to longer ROH segments (>16 Mb).

**Discussion:**

These findings underscore the complexity of inbreeding depression and highlight the importance of considering both the extent and historical depth of inbreeding when evaluating its effects on various traits. Overall, this research provides valuable insights into the genetic basis of inbreeding depression in the Brazilian Angus population and demonstrates the usefulness of genomic data in understanding and mitigating the impacts of inbreeding in livestock populations.

## 1 Introduction

Since Darwin’s early work in identifying reduced growth in self-fertilized plants compared to offspring from unrelated plants (Darwin, 1875; Darwin, 1876), the impact of inbreeding has been studied in various species, including cattle (Doekes et al., 2019; Nishio et al., 2023), sheep (Justinski et al., 2023), and pigs (Silió et al., 2013). Inbreeding depression refers to a decrease in mean performance due to increased levels of inbreeding (Ferenčaković et al., 2017). This increase in inbreeding levels results from the mating of individuals who share a common ancestor (Pryce et al., 2014). Such mating reduces genetic diversity, increasing homozygosity at the expense of heterozygosity (Villanueva et al., 2021).

Inbreeding coefficient was first defined by Wright (1922) as the correlation between homologous alleles of two gametes that unite to form an individual. Later, Malécot (1948) proposed that this could be interpreted as the probability that two homologous alleles at a given locus are identical by descent. Traditionally, the inbreeding coefficient was estimated based on pedigree information (Tenhunen et al., 2024), where the impact of increasing inbreeding values on various traits could be observed and analyzed for inbreeding depression as a regression (Doekes et al., 2021; Villanueva et al., 2021; Mugambe et al., 2024). With the advent of SNP panels, new inbreeding metrics have been proposed, incorporating the concepts of Malécot (1948) and Wright (1922), and estimating inbreeding based on genomic data. Using genomic data allows for a more accurate evaluation of inbreeding, as it has been shown to be closer to the true inbreeding estimates (Peripolli et al., 2017; Peripolli et al., 2018; Peripolli et al., 2020; Forutan et al., 2018). Additionally, genomic-based metrics do not depend on pedigree depth and reliability of pedigree information (Doekes et al., 2019; Nishio et al., 2023).

Several methods have been proposed to study the impact of inbreeding on different traits using both genomic and pedigree data (e.g., Justinski et al., 2023; Nishio et al., 2023; Mugambe et al., 2024). Methods as the FGRM that estimates inbreeding as the deviation of an individual’s genomic relationship with itself (VanRaden, 2008), or the metrics that measures the excess of homozygous genotypes in the population (FHOM1 and FHOM2), yet metrics that calculates the proportion of the genome found in runs of homozygosity (ROH)—long stretches of homozygous genotypes—indicating autozygosity (Ceballos et al., 2018). However, these methods can yield different results, and there is ongoing debate about which metrics best capture inbreeding effects (Villanueva et al., 2021). This debate is still ongoing due to the variability in genomic inbreeding estimates from differences in method sensitivity to recent *versus* ancient inbreeding, dependence on allele frequency assumptions, and marker densities. As a result, no single metric universally captures inbreeding effects, and their performance varies across populations and datasets.

Managing inbreeding and maintaining genetic diversity are crucial for the sustainability of cattle populations. Studies have shown that the reduction in the genetic diversity of the animals has impacted production systems in traits like fertility (Makanjuola et al., 2021), resistance (Mugambe et al., 2024) and productivity (Lozada-Soto et al., 2021) in diverse types of herds. Intense selection practices, artificial insemination, and embryo transfer have significantly reduced effective population size and genetic diversity, increasing homozygosity (Purfield et al., 2012; Rebelato and Caetano, 2018). Modern breeding practices have led to a marked reduction in effective population size (Ne) and a concurrent increase in genomic inbreeding, reflecting elevated autozygosity levels. This trend highlights the unintended consequences of intense selection and widespread use of elite sires, which can exacerbate inbreeding depression and reduce long-term genetic diversity and adaptability. This reduction in genetic diversity can adversely affect herd health and productivity. Therefore, controlling and exploring genetic diversity is essential, especially because only a subset of animals is used as parents of the next-generation (Gurgul et al., 2016). Genomic characterization of genetic diversity is pivotal for designing effective breeding programs and conservation strategies, ensuring that both productivity and resilience are maintained (Brito et al., 2017; Forutan et al., 2018).

The Brazilian Angus Association was established in 1963 and registers both black and red Angus cattle with no distinctions. The Beef Cattle Improvement Program (Promebo^®^, 2020) performs the genetic evaluation for the Brazilian Angus Association as well as to other cattle breed associations in Brazil. Established in 1974 (Campos et al., 2022), Promebo^®^ initially relied solely on phenotypic and pedigree data. While the program was originally focused on improving growth and carcass quality traits, it has expanded to include traits related to disease resistance (e.g., tick resistance) and adaptability (e.g., hair coat), as well as incorporating genomic data to enhance the accuracy of estimated breeding values (EBVs; Campos et al., 2022; Silveira et al., 2021). However, despite advances in commercial genomic evaluations, no study has yet assessed the impact of pedigree and genomic inbreeding on the traits officially evaluated in the Brazilian Angus cattle population which covers traits related to growth, conformation, meat quality, reproduction, resistance to ectoparasites, and heat stress. Therefore, our goals with this study were to: (1) assess six different metrics of inbreeding coefficients (F_PED_, F_GRM_, F_HOM1_, F_HOM2_, F_UNI_ and F_ROH_) in the Brazilian Angus population, which were based on both pedigree and genomic information; and (2) estimate the inbreeding depression for all twelve growth, carcass and adaptation traits currently recorded by the Brazilian Angus Association.

## 2 Materials and methods

Approval from the Animal Care Committee was not needed for this study as all analyses were performed using pre-existing databases.

### 2.1 Genotypes, phenotypes, and pedigree

Genomic information from 13,777 Brazilian Angus animals born between 1988 and 2023 was provided by the Brazilian Angus Association (Promebo^®^, 2020). Animals were genotyped using fourteen different SNP panels with densities ranging from 35 K to 150 K. After combining the SNPs and performing quality control, they were imputed to a density of 78,837 markers using the FImpute software version 3 (Sargolzaei et al., 2014). The reference population for genotype imputation consisted of 835 Brazilian Angus animals born before 2013 and genotyped with high-density SNP panels (150 k SNPs). These animals were selected for their pedigree and phenotypic data to capture the genetic diversity of the population. A rigorous quality control process was applied prior to genotype imputation to ensure data reliability. Samples with low call rates (<90%), extreme heterozygosity, sex mismatches, or duplicates were removed. SNP filtering retained only autosomal markers with CR >98%, MAF >0.03, and Hardy–Weinberg p-values >10^−7^, while redundant or highly correlated SNPs were excluded. Details about the imputation process performed in this population can be found in Campos et al. (2022).

In this study, genotypic quality control procedures were implemented in accordance with the analyses performed. For instance, for the identification of ROH, only data with call rate exceeding 95% for both animals and genotypes were used (no animals or SNP markers were excluded from the analysis based on these criteria). For the other inbreeding metrics used in this study, minor allele frequency (MAF <0.05) and Hardy-Weinberg equilibrium (HWE <10^−6^) were also used to filter out markers. Ultimately, 78,837 and 71,457 SNP autosomal markers were retained for ROH identification and other analyses, respectively. A principal component analysis referring to the population structure is presented in the Supplementary File 1.

Phenotypic information on birth weight, weaning weight gain, weaning conformation, weaning hair coat, post-weaning weight gain, yearling conformation, yearling hair coat, scrotal circumference, ribeye area, subcutaneous back fat thickness, rump fat thickness, intramuscular fat, and tick count were used for this study. Birth weight was measured right after birth (kg). Weaning weight gain was adjusted to 205 days (kg) and post-weaning weight gain was adjusted to 365 days (kg). Weaning conformation and yearling conformation scores are visual measures of the volume of the carcass, taking into account body length and rib depth. Each animal was assigned a score between 1 and 5, with five being the highest expression of the trait and one being the lowest, relative to its contemporary group. Weaning hair coat and yearling hair coat scores range from 1 to 3, with one indicating a short hair coat, two indicating a medium hair coat, and three indicating a long hair coat. Ribeye area (cm^2^), subcutaneous back fat thickness (mm), and intramuscular fat (%) were measured using an ultrasound device on the region between the 12th and 13th ribs, transversely over the *Longissimus dorsi* muscle. Rump fat thickness (mm) was measured by ultrasound on the animal’s rump, between the *Gluteus medius* and *Biceps femoris* muscles. Tick counts were performed manually by counting adult female ticks with at least 4.5 mm on one side of each animal (Wharton et al., 1970). One to three subsequent tick counts were obtained between 2012 and 2022. Tick count records were log-transformed to normalize the distribution, and a constant of 1.001 was added to the counts prior to this transformation because log10(1.0) = 0.0 (Cardoso et al., 2015). Scrotal circumference was measured at yearling, using a specific metal tape at the widest point of the scrotum (cm). Contemporary groups were formed by animals from the same farm, sex, year and season of birth, management group, and date of phenotypic evaluation. Data consistency was ensured by checking for outliers in the continuous traits, i.e., data exceeding 3.5 SD above or below the contemporary group average were excluded. Moreover, contemporary groups with fewer than three animals and/or with no variability for the score traits (i.e., weaning conformation, weaning hair coat, yearling conformation, and yearling hair coat) were also removed (Campos et al., 2022). Descriptive statistics for all traits were computed for both the entire population and a subgroup of only genotyped animals (Table 1).

**TABLE 1:** Descriptive statistics of the traits evaluated in the Brazilian Angus population considering all and only genotyped individuals.

	All					Genotyped				
	*n*	min	max	Mean	sd	*n*	min	max	mean	sd
Birth weight (kg)	243,791	15.00	60.00	33.63	5.08	10,586	15.00	58.00	34.00	5.53
Weaning weight gain (kg)	289,151	20.50	410.00	140.61	39.29	10,089	41.12	381.76	172.00	38.74
Weaning conformation score	273,028	1.00	5.00	3.18	1.09	10,299	1.00	5.00	3.57	1.05
Weaning hair coat	112,757	1.00	3.00	2.01	0.71	8,393	1.00	3.00	1.88	0.71
Post-weaning weight gain (kg)	187,543	0.74	690.00	153.25	76.55	7,894	2.73	563.27	202.69	92.50
Yearling conformation score	188,113	1.00	5.00	3.25	1.07	8,437	1.00	5.00	3.62	1.02
Yearling hair coat	92,376	1.00	3.00	1.81	0.70	7,896	1.00	3.00	1.68	0.70
Scrotal circumference (cm)	50,036	18.00	50.00	34.58	3.69	3,911	21.00	50.00	36.76	3.53
Ribeye area (cm^2^)	30,680	15.06	129.70	59.24	17.90	5,578	15.06	129.70	68.06	17.68
Subcutaneous fat thickness (mm)	29,665	0.10	19.60	2.93	1.58	5,572	0.30	19.00	3.52	1.91
Rump fat thickness (mm)	27,656	0.10	23.90	3.44	2.17	5,574	0.30	18.80	4.23	2.55
Intramuscular fat (mm)	24,450	0.30	8.76	3.02	1.13	5,499	0.49	8.37	2.98	1.14
Tick count (-log10)	6,032	0.00	2.63	1.55	0.42	5,573	0.00	2.63	1.56	0.42

n, number of individuals; min, minimum value for the trait; max, maximum value for the trait; mean, average value for the trait; sd, standard deviation for the trait.

The pedigree information comprises data on 530,327 animals born from 1900 to 2024, with 84,625 classified as founders, 12,069 identified as sires, and 172,602 identified as dams. The longest recorded ancestral path extends up to the 17th generation, with a total of 121,039 animals showing signs of inbreeding. A complete and comprehensive analysis of the pedigree structure is added in the Supplementary File 2.

### 2.2 Runs of homozygosity detection

The PLINK v1.90 software (Purcell et al., 2007) was used to identify ROHs, following the parameters proposed by Mulim et al. (2022). For instance, ROHs were defined using a sliding window of 50 SNPs, a minimum of 30 consecutive SNPs, and a minimum length of 500 kb were required for the region to be identified as ROH. A SNP density of at least 1 SNP per 50 kb was also required, with a maximum gap between consecutive SNPs set at 1,000 kb. A window threshold of 0.05 was applied, allowing for one heterozygous and one missing SNP in the window. After identification, the ROHs were categorized into groups based on length: <2 Mb, 2–4 Mb, 4–8 Mb, 8–16 Mb, and >16 Mb.

### 2.3 Inbreeding metrics

Six different inbreeding metrics were used in this study. The first metric was derived from the pedigree data (F_PED_). F_PED_ was estimated using the CFC software (Sargolzaei et al., 2006), employing the methods outlined by Meuwissen and Luo (1992). The equation used for the calculation of this inbreeding metric is defined as:

$$F_{{PED}_{i}}=A_{ii}-1$$

where **A**_ii_ is the diagonal element of the relationship matrix between the animals of the population on the tabular method proposed by Lush (2010). The second metric used in this study is based on the genotype additive variance (F_GRM_). Therefore, this approach uses the model proposed by VanRaden (2008) defined as:

$$F_{GRM}=G_{ii}-1$$

where

$$G_{ii}=\frac{ZZ^{\prime }}{\sum_{j=1}^{M}2p_{i}\left(1-p_{i}\right)}$$

where **G**_**ii**_ is the genomic relationship matrix, **Z** is the *n x M* matrix of centered genotypes for n individuals, and p_i_ is the reference allele frequency in the population. The third metric was based on the homozygous genotypes observed and expected (F_HOM1_), as proposed by Li and Horvitz (1953):

$$F_{\mathrm{HOM}1}=\frac{H_{\exp}-H_{obs}}{H_{\exp}}$$

where, H_exp_ is the expected value for homozygous genotypes and H_obs_ is the observed value for the homozygous genotypes. Similar to this method, the fourth metric (F_HOM2_) used in this study was also based on the homozygous genotypes following the proposed by Yang et al. (2011), which was estimated as:

$$F_{\mathrm{HOM}2}=1-\frac{\sum_{i}x_{i}*\left(2-x_{i}\right)}{\sum_{i}2p_{i}\left(1-p_{i}\right)}$$

where x_i_ is the number of reference allele copies of the *i*th SNP, and p_i_ is the reference allele frequency in the population.

Given that all previous metrics strongly relied on genotype allele frequency, an additional metric that tries to minimize the effect of allele frequency on the estimation was also tested. This estimation is based on the correlation between uniting gametes (F_UNI_). This method follows the model proposed by Yang et al. (2010), which is defined as:

$$F_{UNI}=\frac{\left[x_{i}^{2}-\left(1+2p_{i}\right)*x_{i}+2p_{i}^{2}\right]}{2p_{i}\left(1-p_{i}\right)}$$

where x_i_ is the number of the reference allele copies of the *i*th SNP, p_i_ is the reference allele frequency in the population.

The final metric used in this study was based on the sum of the individual lengths of ROH divided by the total length of the autosomal genome (F_ROH_). This approach followed the equation proposed by McQuillan et al. (2008):

$$F_{ROH}=\frac{\sum_{i=i}^{n}f\left({ROH}_{i}\right)}{\sum_{j=1}^{A}h\left(j\right)}$$

where $f\left({ROH}_{i}\right)$ represents the length of the ROH for the individual *i*th, *n* denotes the total homozygous genomic regions of each individual, h(j) is the length of the chromosome *j*th, and A is the number of autosomal chromosomes (A = 29). Furthermore, for each class of ROH (<2 Mb, 2–4 Mb, 4–8 Mb, 8–16 Mb, >16 Mb), inbreeding estimates were computed by dividing the total sum of the length of ROH segments by the total length of the autosomal cattle genome. The four first genomic inbreeding coefficients were calculated using the PLINK v1.9 software (Purcell et al., 2007). The correlation among the different inbreeding metrics was assessed using the Pearson correlation coefficient (Pearson, 1896), which was estimated using the R software (R Development Core Team, 2009).

### 2.4 Inbreeding depression

Inbreeding depression was estimated, for each inbreeding metric, using a single-trait linear model by regressing the phenotypes on the estimated inbreeding coefficient. The following statistical model was used to analyze birth weight, post-weaning weight gain, weaning conformation, and weaning hair score:

y=1μ+Xb+βF+Za+Wm+Cmpe+e

where **y** is the vector of phenotypic records; 1 is a vector of ones; µ is the average of the phenotypes; **b** is the vector of fixed effects including contemporary group (farm-year-season), animal’s sex group, cow’s age in years, and age of the animals in days (linear and quadratic effects) as covariates; **F** is the vector of the inbreeding coefficient (F_PED_, F_GRM_, F_HOM1_, F_HOM2_, F_UNI_, F_ROH_, and different groups of ROH classes); and **β** is the linear regression coefficient for the inbreeding coefficient; **a** is the vector of random additive genetic effects, with **a** ∼ (0,**A** $\sigma_{a}^{2}$ ), where **A** is the numerator of the pedigree-based relationship matrix, and $\sigma_{a}^{2}$ is the additive genetic variance; **m** is the vector of random maternal additive genetic effects (not used for weaning conformation and hair score), with **m** ∼ (0,**A** $\sigma_{m}^{2}$ ), **mpe** is the vector of random maternal permanent environment effect with **mpe** ∼ N (0,**I** $\sigma_{mpe}^{2}$ ), and **e** is the random residual, with **e** ∼ N (0,**I** $\sigma_{e}^{2}$ ), where $\sigma_{e}^{2}$ is the residual variance. The **X, Z, W**, and **C** are the incidence matrices related to **b, a, m**, and **mpe**, respectively.

For yearling conformation, yearling hair coat, scrotal circumference, ribeye area, subcutaneous fat thickness, rump fat thickness, and intramuscular fat, the following statistical model was used in the analysis:

y=1μ+Xb+βF+Za+e

where all terms and assumptions were previously defined. For tick count records, which were log-transformed to normalize the distribution, the following model was used:

y=1μ+Xb+βF+Za+Wpe+e

where **pe** is the vector of permanent environmental effects, with **pe** ∼ N (0,**I** $\sigma_{pe}^{2}$ ), where $\sigma_{pe}^{2}$ is the permanent environmental variance; and **W** is the incidence matrix related to **pe**. All other terms and assumptions were previously defined. Confidence intervals for the β coefficients were calculated as $\beta \pm 1.96\times se\left(\beta \right);$ and used to recognize which traits had an inbreeding depression statistically different from zero. Variance components estimation was performed using the Average Information Restricted Maximum Likelihood (AIREML) algorithm implemented in the BLUPF90 family programs (Misztal et al., 2014; Lourenco et al., 2020).

## 3 Results

### 3.1 Inbreeding coefficients

The summary of the inbreeding coefficients estimated using the different metrics evaluated in this study is shown in Table 2.

**TABLE 2:** Summary of different inbreeding coefficient metrics in the Brazilian Angus population.

	*n*	min	max	Mean	sd
F_PEDtotal_	530,263	0.00	0.38	0.01	0.02
F_PEDinbreed_	121,039	0.00	0.38	0.02	0.04
F_PEDgen_	13,777	0.00	0.28	0.01	0.02
F_GRM_	13,777	−0.16	0.71	0.01	0.06
F_HOM1_	13,777	−0.32	0.22	0.01	0.04
F_HOM2_	13,777	−0.33	0.23	0.01	0.04
F_UNI_	13,777	−0.23	0.35	0.01	0.03
F_ROH_	13,777	0.00	0.32	0.13	0.03
<2 Mb	13,777	0.00	0.05	0.01	0.00
2–4 Mb	13,777	0.00	0.09	0.03	0.01
4–8 Mb	13,777	0.00	0.10	0.04	0.01
8–16 Mb	13,777	0.00	0.10	0.03	0.01
>16 Mb	13,777	0.00	0.21	0.02	0.02

n, number of individuals; min, minimum individual inbreeding coefficient; max, individual inbreeding coefficient; mean, average of the inbreeding coefficient; sd, standard deviation of the inbreeding coefficients; F_PEDtotal_, inbreeding coefficient using the entire pedigree information; F_PEDinbreed_, inbreeding coefficient using only the inbreed animals from pedigree information; F_PEDgen_, inbreeding coefficient based on pedigree for the genotyped animals; F_GRM_, inbreeding coefficient based on the genotype additive variance; F_HOM1_, inbreeding coefficient based on the homozygous genotypes observed and expected; F_HOM2_, inbreeding coefficient based on homozygous genotypes; F_UNI_, inbreeding coefficient based on the correlation between uniting gametes; F_ROH_, inbreeding coefficient based on the length of the ROH’s and the total length of the autosomal genome.

**FIGURE 1:**
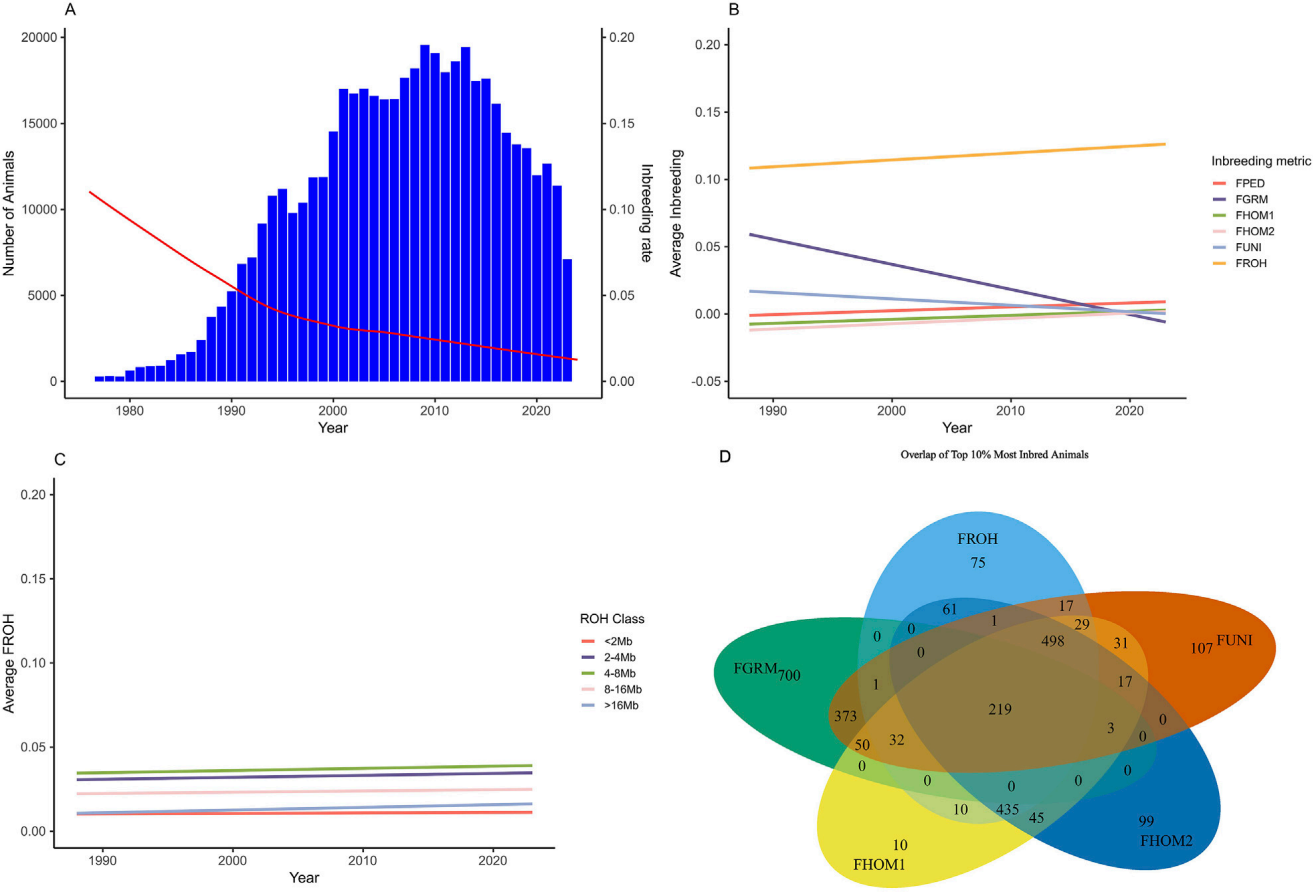
Trend of average population inbreeding over the year for the Brazilian Angus Population and the overlap of the top 10% most inbred animals using genomic dats. **(A)** Trend of the average pedigree inbreeding (red line) and number of individuals in the population (blue columns). **(B)** Average inbreeding coefficient for the genotyped population over the years for the different metrics of inbreeding evaluated. **(C)** Average inbreeding coefficient for the genotyped population over the years based on the classes of runs of homozygosity (FROH). **(D)** Venn diagram display the overlapping of the top 10% of the most inbreed animals for which genomic metric. FPED, inbreeding coefficient based on pedigree for the genotyped animals; FGRM, inbreeding coefficient based on the genotyped additive variance; FHOM1, inbreeding coefficient based on the homozygous genotyped observed and expected; FHOM2, inbreeding coefficient based on homozygous genotyped; FUNI, inbreeding coefficient based on the correlation between uniting gametes; FROH, inbreeding coefficient based on the length of the ROH’s and the total length of the autosomal genome.

Individual inbreeding coefficients ranged from −0.33 to 0.71, using the different metrics (Table 2). The metrics F_PEDtotal_, F_PEDgen_, F_GRM_, F_HOM1_, F_HOM2_, F_UNI_, and the class of <2 MB of the ROH had the lowest average inbreeding coefficient (0.01). On the other hand, the F_ROH_ metric showed the highest inbreeding coefficient (0.13 ± 0.03). Figure 1 illustrates the average inbreeding over time based on pedigree data, alongside the number of animals in the pedigree file (Figure 1A). Additionally, it shows the trend of average inbreeding population over the years for the genotyped animals using different inbreeding metrics (Figures 1B,C) and the overlap of the 10% most inbred animals for the genomic inbreeding metrics (1D).

**FIGURE 2:**
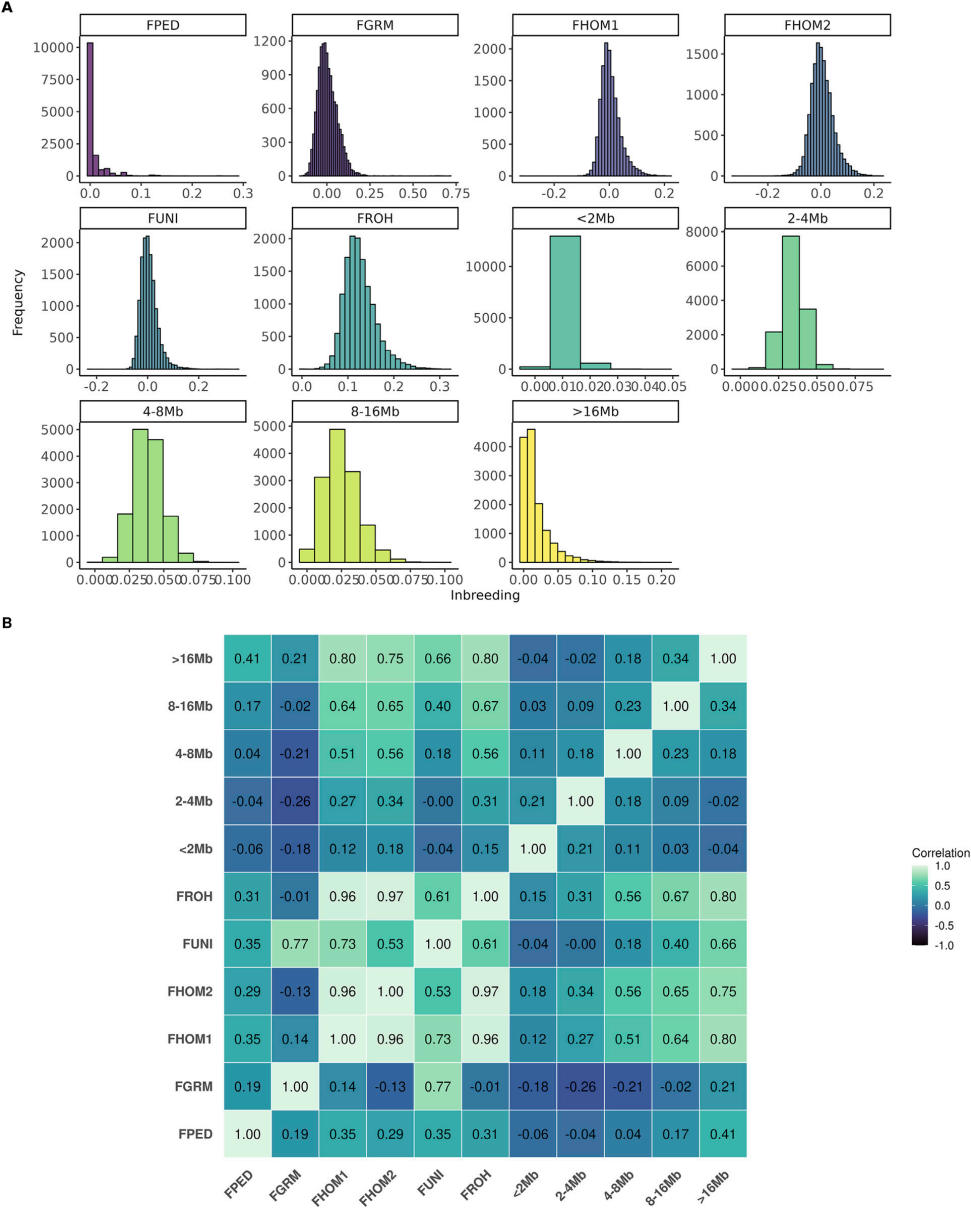
Distribution frequency **(A)** and correlation among the inbreeding metrics **(B)** evaluated for the Brazilian Angus population. FPED, inbreeding coefficient based on pedigree for the genotyped animals; FGRM, inbreeding coefficient based on the genotyped additive variance; FHOM1, inbreeding coefficient based on the homozygous genotyped observed and expected; FHOM2, inbreeding coefficient based on homozygous genotyped; FUNI, inbreeding coefficient based on the correlation between uniting gametes; FROH, inbreeding coefficient based on the length of the ROH’s and the total length of the autosomal genome.

In general, the number of animals in the population increased until 2013, followed by a decline. Conversely, the average of inbreeding, based on pedigree, decreased during this period (Figure 1A). On the other hand, as shown in Figure 1B, the average inbreeding for the genotyped animals increased over the years according to the F_ROH_, F_PED_, F_HOM1_, and F_HOM2_ metrics, while the F_GRM_ and F_UNI_ metrics showed a decrease. Figure 2 shows the distribution frequency of inbreeding metrics and the correlations among the various inbreeding metrics.

The correlation among different inbreeding metrics ranged from weak (−0.26; between F_GRM_ and F_2–4Mb_) to strong (0.97; between F_ROH_ and F_HOM2_). The metrics that had high correlations among themselves were F_HOM1_, F_HOM2_, F_UNI_, and F_ROH_. The F_PED_ metric showed a weak correlation with all the others, with its highest correlation being observed with the segments of ROH longer than 16 Mb (i.e., 0.41). Negative correlations were observed among F_GRM_ and the majority of inbreeding coefficients derived from the ROH segments.

### 3.2 Inbreeding depression

Table 3 shows the estimates of inbreeding depression for all traits analyzed in this study, using the different inbreeding metrics. Interestingly, the extent of inbreeding depression varied across the traits evaluated (Table 3). While significant effects were evident for several key growth and carcass component traits, other traits such as tick count did not exhibit statistically significant impact from inbreeding depression.

**TABLE 3:** Estimates of inbreeding depression for all traits analyzed in this study, using different inbreeding metrics.

Trait	F_PEDtotal_	F_PEDgen_	F_GRM_	F_HOM1_	F_HOM2_	F_UNI_	F_ROH_	2 Mb	2–4 Mb	4–8 Mb	8–16 Mb	>16 Mb
Birth weight (kg)	−0.01%	−0.14%^*^	−0.07%^*^	−0.07%	−0.06%	−0.09%^*^	−0.08%	0.83%*	−0.24%	0.04%	−0.22%^*^	−0.09%
Weaning weight gain (kg)	−0.12%^*^	−0.12%	−0.18%^*^	−0.24%^*^	−0.21%^*^	−0.27%^*^	−0.26%^*^	0.05%	−0.29%	−0.15%	−0.42%^*^	−0.29%^*^
Weaning conformation score	−0.15%^*^	−0.40%^*^	−0.28%^*^	−0.26%^*^	−0.21%^*^	−0.36%^*^	−0.25%^*^	0.79%	0.17%	−0.06%	−0.41%	−0.38%^*^
Weaning hair coat	0.13%^*^	0.33%	0.06%	0.23%	0.22%	0.19%	0.23%	1.51%	0.31%	0.54%	0.50%	0.08%
Post-weaning weight gain (kg)	−0.22%^*^	−0.39%^*^	−0.20%^*^	−0.43%^*^	−0.40%^*^	−0.42%^*^	−0.45%^*^	−0.50%	−0.28%	−0.82%^*^	−0.64%^*^	−0.47%^*^
Yearling conformation score	−0.28%^*^	−0.41%^*^	−0.30%^*^	−0.47%^*^	−0.43%^*^	−0.52%^*^	−0.49%^*^	0.77%	−0.15%	−0.56%	−1.00%^*^	−0.53%^*^
Yearling hair coat	0.15%^*^	0.20%	0.01%	0.26%	0.26%	0.18%	0.32%	1.24%	1.44%^*^	0.28%	0.76%^*^	0.14%
Scrotal circumference (cm)	−0.08%^*^	−0.20%^*^	−0.15%^*^	−0.12%^*^	−0.09%^*^	−0.17%^*^	−0.14%^*^	0.24%	−0.04%	−0.08%^*^	−0.16%	−0.20%^*^
Ribeye area (cm^2^)	−0.23%^*^	−0.27%^*^	−0.23%^*^	−0.33%^*^	−0.29%^*^	−0.36%^*^	−0.34%^*^	−0.84%	−0.21%	−0.48%	−0.37%^*^	−0.40%^*^
Subcutaneous fat thickness (mm)	−0.15%	−0.48%^*^	−0.32%^*^	−0.50%^*^	−0.44%^*^	−0.54%^*^	−0.45%^*^	1.32%	−0.55%	−0.70%	−0.59%	−0.51%^*^
Rump fat thickness (mm)	−0.30%^*^	−0.29%	−0.11%	−0.61%^*^	−0.62%^*^	−0.48%^*^	−0.68%^*^	1.12%	−0.88%	−0.71%	−1.13%^*^	−0.72%^*^
Intramuscular fat (mm)	0.19%	0.10%	0.01%	0.30%	0.33%^*^	0.21%	0.35%	1.24%	0.51%	0.89%	0.66%	0.18%
Tick count (−log10)	0.00%	−0.01%	0.00%	0.00%	0.00%	0.00%	0.00%	0.05%	0.02%	−0.01%	−0.01%	−0.01%

The percentages represent the impact on each trait for a 1% increase in inbreeding.

F_PEDtotal_, inbreeding coefficient using the entire pedigree information; F_PEDgen_, inbreeding coefficient based on pedigree for the genotyped animals; F_GRM_, inbreeding coefficient based on the genotyped additive variance; F_HOM1_, inbreeding coefficient based on the homozygous genotyped observed and expected; F_HOM2_, inbreeding coefficient based on homozygous genotyped; F_UNI_, inbreeding coefficient based on the correlation between uniting gametes; F_ROH,_ inbreeding coefficient based on the length of the ROH’s and the total length of the autosomal genome.

*Inbreeding depression statistically different from zero.

The estimation of inbreeding depression on the traits analyzed in this study showed a predominantly unfavorable effect, with increased inbreeding having the most significant unfavorable impact on rump fat thickness, where a 1% increase in the inbreeding coefficient decreases the performance of the trait up to 1.13%, considering the F_8–16Mb_ metric. Conversely, favorable associations with increasing inbreeding were observed for intramuscular fat (F_HOM2_) and birth weight (F_2Mb_). In summary, the inbreeding depression on different traits ranged from −1.13% in the F_8–16Mb_ metric for rump fat thickness to 0.83% in the F_2Mb_ metric for birth weight. The b values and their respective standard deviations can be found in Supplementary File 3.

## 4 Discussion

In this study, our goal was to assess different metrics of inbreeding and their impact on various traits currently evaluated in the Brazilian Angus population. Differences in the average inbreeding by year measured by the different metrics were observed (Table 2; Figure 1). This difference is somehow expected, as each metric captures different concepts of inbreeding occurring in the population. For example, the inbreeding based on the pedigree estimates derived from Meuwissen and Luo (1992) take into account the covariance relationship matrix and estimate the expected proportions of genes shared by individuals in the pedigree. This metric depends on the relationship among individuals and the connections within the pedigree records (Baes et al., 2019), as well as the quality and number of recorded generations. As a result, individuals considered as founders of the population are assumed to be unrelated, which can bias the estimates when compared to the use of genomic information. This assumption may explain the general trend observed of decreasing average inbreeding over time when using the pedigree information (Figure 1A). The increasing population size indicates that the introduction of individuals unrelated to the original pedigree (or without complete pedigree information) has occurred.

For the genotyped population, different trends were observed for various metrics (Figure 1B), showing divergence in measuring inbreeding based on the concepts of Wright (1922) and Malécot (1948). For instance, the F_GRM_ metric showed a decrease over time in the inbreeding coefficient, even reaching slightly negative values. These negative values indicate an increase in genetic variability compared with the initial population (Muñoz et al., 2019; Villanueva et al., 2021; Mulim et al., 2022a). This method relies on allele frequencies and may not function correctly if these frequencies differ from those in the founder population (Nishio et al., 2023). This can also affect the correlation between metrics. At the regional genomic level, frequency changes can be more pronounced, leading to lower correlations—or even negative ones—between coefficients (Villanueva et al., 2021), as seen in the case of F_GRM_. Additionally, this metric assigns greater weight to rare alleles, meaning that an individual homozygous for a rare allele will have a higher inbreeding coefficient than one homozygous for a common allele (Alemu et al., 2021; Villanueva et al., 2021). This introduces a bias in the information and does not accurately reflect the true dynamics of the population.

The same issue affects the F_HOM1_ and F_HOM2_ metrics (Table 2; Figure 1B), which measure the reduction in heterozygosity. The dependency on allele frequency can influence the results (Howard et al., 2017). To address this, we also analyzed the F_UNI_ model, which is based on the principle proposed by Wright (1922), where inbreeding is measured as the correlation between parental gametes. One advantage of the F_UNI_ metric is its ability to capture variation in inbreeding due to distant ancestors (Kardos et al., 2018). However, this metric is more suitable for scenarios involving large population sizes, such as human populations (Polak et al., 2021).

Regarding F_ROH_, this metric shows the highest inbreeding coefficient in the population and an increase over time. It captures not only recent inbreeding but also inbreeding from more distant generations (Ceballos et al., 2018). For instance, Howrigan et al. (2011) and Tenhunen et al. (2024) demonstrated that ROHs shorter than 2 Mb were likely created between 25 and 50 generations ago, while ROHs around 16 Mb in length were estimated to have been created about three generations ago. This distinction helps identify when inbreeding occurred, as well as assessing the impact of inbreeding. More recent inbreeding can have more negative effects compared to ancient inbreeding (Doekes et al., 2019). This was also observed in our study, where different classes of inbreeding had varying impacts on traits (Table 3). In general, the inbreeding coefficient estimated from shorter ROH segments (e.g., <2 Mb) had a smaller and sometimes even beneficial effect compared to the inbreeding coefficient estimated from longer ROH segments (e.g., >16 Mb).

These differences also influence the correlation among the metrics (Figure 2). For instance, F_HOM1_, F_HOM2_, F_UNI_, and F_ROH_ have a high correlation with each other, while F_GRM_ has a low correlation with these metrics. Interestingly, the stronger correlation estimated between F_PED_ and F_ROH16_ compared to inbreeding estimated from shorter ROHs might indicate that the pedigree is not sufficiently deep in this population to accurately captures ancient inbreeding events. The choice of the optimal metric to estimate inbreeding in a population, as suggested by Alemu et al. (2021), depending on the intended application. For cases with finite and small population sizes, F_ROH_ is considered the best approach for estimating the inbreeding coefficient used to predict inbreeding depression (Alemu et al., 2021; Polak et al., 2021). Moreover, for studies aiming to understand the impact of recent inbreeding in the inbreeding depression, F_ROH_ may also be the most informative, as it has the ability to distinguish between ancient and recent inbreeding easily. On the other hand, F_ROH_ depends on the density and quality of genotyped panels, as well as the parameters selected to estimate ROH (Mulim et al., 2022a). Since there is still no clear consensus in the literature on the optimal parameters for identifying ROH in the animal genome, the results may be subject to bias.

Similar inbreeding depression for birth weight, weaning weight, and post-weaning gain was found in the American Angus population using pedigree and genomic metrics (Lozada-Soto et al., 2021). However, there is a gap in the literature regarding the impact of inbreeding on other traits in the Angus population. Notably, tick count is the only trait that seems not to be affected by inbreeding (Table 3; Supplementary File 3). This might be related to the fact that tick count had the smallest number of animals with phenotypes in our study (*n* = 2,765), potentially decreasing the statistical power of the analysis for this trait. The differential sensitivity of traits to inbreeding is consistent with broader literature, often reflecting differences in their genetic architecture, heritability, and historical selection pressures. Traits with lower heritability or those more closely linked to overall fitness (though direct fitness traits were not assessed here) can sometimes exhibit more pronounced inbreeding depression. The lack of significant depression for tick count might suggest that either the current levels of inbreeding have not yet critically impacted the loci governing this trait, or that other environmental and management factors play a more dominant role in its expression within this population. Nonetheless, the impact of inbreeding depression on tick count warrants further investigation, as parasite resistance is a crucial adaptive trait in tropical environments.

A favorable impact on inbreeding depression for shorter ROHs (F_2Mb_) and an unfavorable one for longer ROHs (F_8–16Mb_) was also observed for birth weight. This differential impact across ROH classes exemplifies the nuanced relationship between inbreeding and trait performance. The observation highlights that while shorter ROHs may reflect more ancient and manageable levels of inbreeding, longer ROHs, indicative of recent or more extensive inbreeding, tend to have more detrimental effects. These findings emphasize the complexity of inbreeding depression and underscore the importance of considering both the length and historical depth of inbreeding when evaluating its effects on economically important traits like birth weight. These findings have direct implications for genetic improvement programs in Brazilian Angus cattle. The clear evidence of inbreeding depression for economically relevant traits necessitates the implementation of strategies aimed at monitoring and controlling the rate of inbreeding accumulation. The use of genomic inbreeding coefficients, such as FROH, as demonstrated in this study (Table 3), provides a powerful tool for accurately assessing individual autozygosity and identifying animals at higher risk. To mitigate the adverse effects of inbreeding, several management strategies can be employed. Optimal contribution selection, which aims to maximize genetic gain while constraining the rate of inbreeding, is a key approach. Furthermore, genomic mating programs can be designed to allocate matings that minimize the expected FROH in offspring, thereby directly addressing the accumulation of deleterious recessive alleles. Careful management of popular sires to prevent their overuse and maintain broader genetic diversity within the breed is also essential.

The intensity of the impact of inbreeding depression depends on the type of allelic interaction involved (Carr and Dudash, 2003) and the presence of possible deleterious mutations (Charlesworth and Willis, 2009). Additionally, changes in mean phenotypic performance due to inbreeding are influenced by the frequency of alleles in homozygous states (Doekes et al., 2020). These concepts highlight the need for further studies that account for these factors in ROH association analyses. By doing so, researchers can better capture the magnitudes of inbreeding depression at specific loci, leading to a more precise understanding of how inbreeding affects various traits. This approach can help identify critical loci that contribute to inbreeding depression and improve strategies for managing genetic diversity in populations.

### 4.1 Limitations and perspectives

The accurate quantification of inbreeding is fundamental for developing effective breeding designs strategies, as it directly impacts the interpretation and management of inbreeding depression. While traditional pedigree-based inbreeding coefficients (FPED) provide estimates based on recorded ancestry, they can be compromised by errors or incompleteness in pedigree data, potentially overlooking cryptic relatedness, particularly in populations with shallow or poorly documented relationships. In comparison, genomic-based metrics, such as FROH, offer a more precise alternative by directly estimating realized autozygosity from an individual’s genome. This approach provides higher resolution and greater power to detect recent inbreeding events or segmental autozygosity. Among genomic metrics, we believe FROH is particularly valuable due to its ability to distinguish between recent and ancient inbreeding. For instance, our results suggest that longer ROH segments, indicative of recent inbreeding, seems to be associated with more severe inbreeding depression. Thus, we believe that for producers and breeding programs, focusing on minimizing the accumulation of long ROH segments might be a pragmatic initial approach, while still monitoring overall FROH from various length classes to manage long-term genetic diversity.

Despite the valuable insights this study offers regarding inbreeding effects on key traits in Brazilian Angus cattle, several limitations warrant consideration. The accuracy of genomic inbreeding coefficients is highly dependent on factors such as SNP panel density, marker distribution, and overall genotyping quality. In this context, we acknowledge that genotype imputation, while a valuable tool, can introduce certain limitations and potential biases in our study. For instance, the underestimation of rare variants or the potential for imputation errors to slightly affect downstream analyses like ROH calling, particularly for very short segments. We emphasize that while we employed best practices to minimize such issues, the inherent limitations of imputation should be considered when interpreting the results of this paper. Additionally, the availability of sparse phenotypic data for certain traits, for example, tick count, reduced the statistical power for detecting inbreeding depression. It is also important to note that the analytical models used in this study did not explicitly account for the individual effects of deleterious recessive alleles or complex allele interactions, such as dominance or over dominance, which can likely modulate the expression of inbreeding depression.

The integration of genomic inbreeding metrics into breeding programs offers a powerful complement or alternative to traditional pedigree-based approaches, particularly in populations like Brazilian Angus, where pedigree information may be incomplete. These metrics enhance the efficiency of advanced strategies such as optimum contribution selection and genomic-based mating allocation, helping breeders balance genetic gain with the control of inbreeding. Moreover, monitoring ROH can identify individuals at risk of inbreeding depression and guide more informed mating decisions, promoting long-term genetic diversity and adaptability under tropical conditions. While our study focused more on carcass and growth traits due to data availability, we acknowledge the critical importance of reproductive traits—highly sensitive to inbreeding depression—and recommend that future research prioritize their inclusion to fully assess the impact of inbreeding and refine genetic management strategies.

## 5 Conclusion

This study provides valuable insights into the effects of inbreeding on various traits within the Brazilian Angus population, using several inbreeding metrics based on both pedigree and genomic information. Our findings highlight significant variations in inbreeding coefficients across different metrics, with the highest values observed in the F_ROH_ metric, indicative of its capacity to capture both recent and distant inbreeding. This divergence underscores the importance of metric selection in inbreeding studies, where each metric provides unique insights based on its underlying assumptions and methodologies. Inbreeding depression analysis revealed predominantly unfavorable effects of inbreeding on several traits, with notable impacts on rump fat thickness. Conversely, some traits, such as intramuscular fat and birth weight, showed favorable associations with inbreeding at low levels, suggesting a complex relationship between inbreeding and phenotypic expression that varies across different traits. This complexity is further reflected in the varying impacts observed across different ROH classes, where shorter ROH segments often exhibited smaller or even beneficial effects compared to longer segments.

## Data Availability

The data analyzed in this study is subject to the following licenses/restrictions: The datasets are property of the PROMEBO Beef Cattle Breeding Program producers and Embrapa, and this information is commercially sensitive. Requests to access these datasets should be directed to fernando.cardoso@embrapa.br.
