# Supplementary figures and images for: Exploring inbreeding depression in Brazilian Angus cattle population using pedigree and genomic data
Source: Front Genet. 2025 Jun 9;16:1613820. doi: 10.3389/fgene.2025.1613820 (PMC12183215; doi:10.3389/fgene.2025.1613820)

PCA of Angus populations

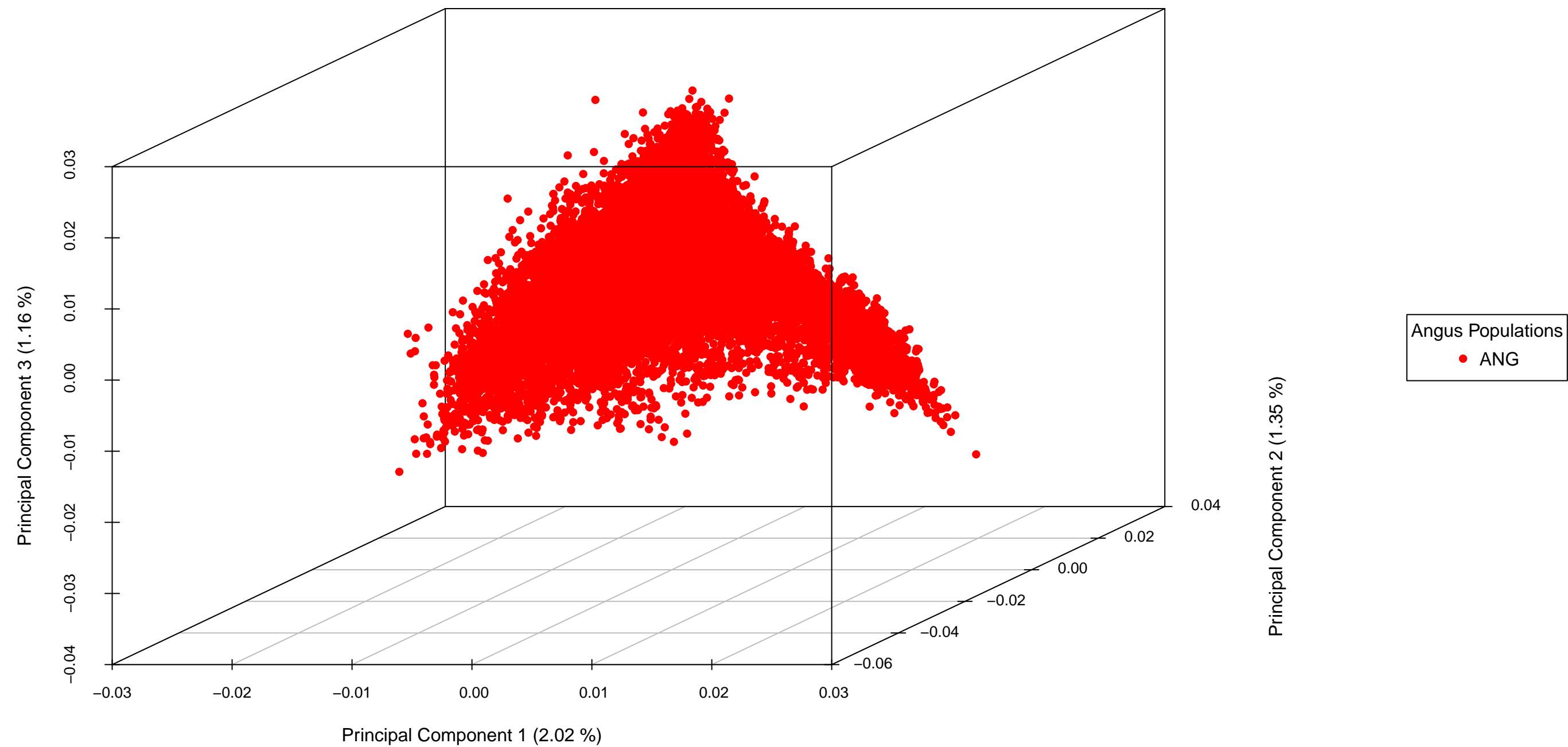

Supplement: Supplementary file 1 [file Supplementaryfile1.pdf]
